# Supplementary material for: Spatial clusters of dominant lineages of uropathogenic Escherichia coli in a community dwelling patient population
Source: BMC Infect Dis. 2025 Oct 21;25:1379. doi: 10.1186/s12879-025-11734-4 (PMC12542483; doi:10.1186/s12879-025-11734-4)
Supplement: Supplementary file 1 — Supplementary Material 1. [file 12879_2025_11734_MOESM1_ESM.docx]

| **Primer** | **Sequence Type** | **Sequence (5’-3’)** | **Expected product size (bp)** |
| --- | --- | --- | --- |
| ST69_for | 69 | ATCTGGAGGCAACAAGCATA | 104 |
| ST69_rev |  | AGAGAAAGGGCGTTCAGAAT |  |
| ST95_for | 95 | ACTAATCAGGATGGCGAGAC | 200 |
| ST95_rev |  | ATCACGCCCATTAATCCAGT |  |
| ST131_for | 131 | GACTGCATTTCGTCGCCATA | 310 |
| ST131_rev |  | CCGGCGGCATCATAATGAAA |  |
| ST73_for | 73 | TGGTTTTACCATTTTGTCGGA | 490 |
| ST73_rev |  | GGAAATCGTTGATGTTGGCT |  |

Table S1. Primer sequences used in the multiplex PCR assay for the detection of dominant UPEC sequence types. All primer sequences and assay specifications were referenced from Doumith et. al 2015
